# Polypharmacy and mortality association by chronic kidney disease status: The *RE*asons for *G*eographic *A*nd *R*acial *D*ifferences in *S*troke Study

**DOI:** 10.1002/prp2.823

**Published:** 2021-08-02

**Authors:** Winn Cashion, William McClellan, Suzanne Judd, Abhinav Goyal, David Kleinbaum, Michael Goodman, Valerie Prince, Paul Muntner, George Howard

**Affiliations:** ^1^ Department of Epidemiology Emory University Rollins School of Public Health Atlanta GA USA; ^2^ Department of Biostatistics University of Alabama at Birmingham School of Public Health Birmingham AL USA; ^3^ Department of Pharmacy Practice Samford University McWhorter School of Pharmacy Birmingham AL USA; ^4^ Department of Epidemiology University of Alabama at Birmingham School of Public Health Birmingham AL USA

**Keywords:** chronic kidney disease, epidemiology, mortality, polypharmacy, REGARDS cohort study

## Abstract

Many Americans take multiple medications simultaneously (polypharmacy). Polypharmacy's effects on mortality are uncertain. We endeavored to assess the association between polypharmacy and mortality in a large U.S. cohort and examine potential effect modification by chronic kidney disease (CKD) status. The *RE*asons for *G*eographic *A*nd *R*acial *D*ifferences in *S*troke cohort data (*n* = 29 627, comprised of U.S. black and white adults) were used. During a baseline home visit, pill bottle inspections ascertained medications used in the previous 2 weeks. Polypharmacy status (major [≥8 ingredients], minor [6–7 ingredients], and none [0–5 ingredients]) was determined by counting the total number of generic ingredients. Cox models (time‐on‐study and age‐time‐scale methods) assessed the association between polypharmacy and mortality. Alternative models examined confounding by indication and possible effect modification by CKD. Over 4.9 years median follow‐up, 2538 deaths were observed. Major polypharmacy was associated with increased mortality in all models, with hazard ratios and 95% confidence intervals ranging from 1.22 (1.07–1.40) to 2.35 (2.15–2.56), with weaker associations in more adjusted models. Minor polypharmacy was associated with mortality in some, but not all, models. The polypharmacy–mortality association did not differ by CKD status. While residual confounding by indication cannot be excluded, in this large American cohort, major polypharmacy was consistently associated with mortality.

## Graphical abstract

Polypharmacy was associated with all‐cause mortality in a dose‐response fashion in the REGARDS cohort. Although having CKD is an independent mortality risk factor, there was no evidence of a greater polypharmacy mortality effect in those with CKD than those without CKD.

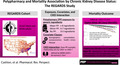

**Abbreviations**
ARIC: atherosclerosis risk in communities cohort
BMI: body mass index
CKD: chronic kidney disease
CVD: cardiovascular disease
Fib: fibrillation
HR: hazard ratio
OTC: over‐the‐counter
REGARDS: *RE*asons for *G*eographic *A*nd *R*acial *D*ifferences in *S*troke Study
SAS: Statistical Analysis Software
SES: socioeconomic status

## 1 INTRODUCTION

Americans consume many prescription and over‐the‐counter (OTC) medications.^1^, ^2^ With over 300 000 marketed OTC products^3^ and approximately 5 billion OTC products purchased annually,^4^ an estimated 70%–90% of illnesses involve at least some self‐treatment.^5^

While medications’ health benefits are indisputable, approximately half of all prescriptions may be used improperly.^6^ Additionally, drugs’ side effects are often treated with more medication, leading to a “prescribing cascade.”^7^ Drug allergies, drug–drug and drug–disease interactions, and direct toxicity are all hazards. If categorized as a disease, adverse drug reactions are estimated to be the fourth leading cause of death.^8^

Polypharmacy, or high medication use,^9^ can exert polytherapeutic effects and/or polytoxicities.^10^ The term “polypharmacy” sometimes has negative connotations, suggesting inappropriate/excessive medication use; however, the simultaneous administration of many drugs can be the standard of care. Polypharmacy is often defined in two ways: using more drugs than clinically warranted or taking more than a threshold drug count, for example, five.^11^

Polypharmacy is a known risk factor for adverse health events, including cognitive decline,^12^, ^13^ falls,^14^, ^15^ and adverse drug reactions.^16^ Based on possible drug–drug interactions^17^ and adverse drug reactions,^16^ polypharmacy poses plausible risks; however, the relation of polypharmacy with mortality among general, community‐dwelling Americans remains largely unexplored. Individuals with chronic kidney disease (CKD) may be especially vulnerable to any adverse effects of polypharmacy because kidney function is critical for drug excretion; however, data are very limited on CKD’s role in the polypharmacy–mortality association. Addressing these knowledge gaps, we analyzed the large, national *RE*asons for *G*eographic *A*nd *R*acial *D*ifferences in *S*troke cohort.

## 2 MATERIALS AND METHODS

### 2.1 Study design

*RE*asons for *G*eographic *A*nd *R*acial *D*ifferences in *S*troke cohort is a nationwide, longitudinal study that began in 2003 and was described previously.^18^ Briefly, the analytic sample consisted of 29 627 (Data S1) community‐dwelling black and white Americans age ≥45 years with at least one follow‐up mortality assessment. The cohort recruitment occurred throughout the continental United States using the Genesys commercial database,^19^ with oversampling of blacks and “stroke belt”^20^ residents (eight Southeastern states: NC, SC, GA, TN, AL MS, AR, and LA). The Institutional Review Board reviewed the research at Emory University and the University of Alabama Birmingham.

Individuals were excluded from the cohort for non‐black/non‐white race, ongoing cancer treatment, inability to speak English, nursing home residence, telephone interviewer‐assessed cognitive impairment, or if expected to pose follow‐up difficulties. The cohort's cooperation rate was 49%.^21^

### 2.2 Data

A computer‐assisted telephone interview collected information on demographic, socioeconomic status (SES), medical, and lifestyle variables. Examination Management Services Inc. scheduled a home visit and instructed the participant to collect all medicines used in the previous 2 weeks. During the home visit, signed informed consent was obtained, and anthropomorphic measurements and blood samples were collected and sent to a central laboratory. The company's personnel examined each medicine present (“pill bottle” inspection including creams/eye drops/injectables) and cataloged its name (generic/brand), but neither dose nor use frequency, on a standardized form. Medications given outside the home, such as at an infusion center, were not included. These records were processed into an electronic database of 34 776 distinct recorded medication names. For prescriptions/OTCs, a generic name and medication class were assigned (e.g., acetaminophen, miscellaneous analgesic) by a research pharmacist and project staff using *Drugs*.*com*.^22^ For 1.62% of medications, the generic name could not be identified (e.g., “amocardone” or “tylewok”) and were assigned the generic name “unknown”. Each unknown was assumed to correspond to one generic ingredient.

When assessing polypharmacy, supplements (vitamins/minerals/herbals/nutraceuticals) were excluded due to their heterogeneity, lack of universal nomenclature, and limited US Food & Drug Administration oversight.^23^, ^24^ Some vitamins/minerals are available both in supplemental and prescription varieties; we tried to distinguish the prescription forms which counted toward polypharmacy (e.g., isotretinoin) from the OTC‐available forms (e.g., vitamin A) that were considered supplements. Many drugs come in combination form; the combination pill generic ingredient count was the total number of active ingredients. Some participants reported taking the same generic drug multiple times, whether from different formulations (e.g., long‐, medium‐, and short‐acting insulin) or using the same medicine twice (e.g., two different acetaminophen‐containing, multicomponent analgesics); in such cases, the total ingredient sum included that agent multiple times.

Polypharmacy was characterized using three categories of total prescription/OTC medication ingredient counts (excluding supplements), as suggested elsewhere^25^: no polypharmacy (≤5 total ingredients), minor polypharmacy (6–7 ingredients), and major polypharmacy (≥8 ingredients). Presence of CKD was defined as self‐reported dialysis or glomerular filtration rate <60 mL/min/1.73 m^2^ using the modified diet in renal disease equation applied to serum creatinine collected with baseline laboratories (albuminuria was not considered).^2^, ^26^

Cohort members were called approximately every 6 months to ascertain vital status. Additionally, deaths were identified through proxy communication and Social Security death index master file and National Death Index checks. During a maximum of over 7 years of follow‐up, fewer than 3% of participants were lost to follow‐up annually. Of the original cohort (*n* = 30 239), 58 (0.2%) had data anomalies or lacked medication data, and 554 (1.8%) lacked any follow‐up vital status or follow‐up time and were excluded from analyses. A total of 2538 deaths (8.6%) were observed through September 2010. Regarding follow‐up completeness, 50% of survivors had vital status ascertained within 115 days of the last recorded follow‐up; 75% within 195 days; and 90% of survivors had vital status ascertained within 2.35 years of the last recorded follow‐up.

### 2.3 Covariates

Known polypharmacy risk factors include comorbidities,^10^, ^27^ activities of daily living dependence,^27^ demographics (female sex,^10^, ^28^ older age,^10^, ^28^ and white race^27^, ^29^), and SES (lower education,^10^, ^28^ lower social status,^10^, ^30^ and unemployment^10^, ^30^). We adjusted for potential confounding using the following full‐model covariates: demographics (age [45–54, 55–64, 65–74, 75–84, 85+ years], race [black, white], sex, relationship status [widowed, divorced, married, single, other], region [stroke buckle (Georgia, North Carolina, and South Carolina coastal plain), stroke belt, non‐belt]); SES measures (education [< or ≥high school] and income [<$20, $20–$34, $35–$74, $75 k+, refused], health insurance [medical care]); lifestyle variables (alcohol [heavy/moderate/none], smoking [current/past/never], body mass index [BMI: categories enumerated in Table 2], physical activity); comorbidities (CKD, diabetes, atrial fibrillation, hypertension, cardiovascular disease, dyslipidemia—all dichotomous; defined in the Data S1); and self‐reported health and stress.

### 2.4 Statistical analysis

Cox proportional hazards models with the time‐on‐study outcome (or attained age outcome^31^, ^32^) until death or censoring examined the polypharmacy–mortality association. CKD was evaluated a priori as a potential effect modifier of polypharmacy on mortality. The age‐time‐scale models included the same covariates, except attained age was instead the outcome of interest (conditioning on study entry age, with birth cohort stratification). Models 1–7 (Table 1) are subsets of the “full” model 8. Multiple models were utilized because the causal pathway for polypharmacy and mortality is not established, particularly given this cohort's heterogeneity. Aside from models 1–7, no other “reduced” models were considered.

**TABLE 1:** Multiple models considered to assess polypharmacy–mortality association

		Mod. 1	Mod. 2	Mod. 3	Mod. 4	Mod. 5	Mod. 6	Mod. 7	Mod. 8
Demographics	Age	X	X	X	X	X	X	X	X
	Region	X	X	X	X	X	X	X	X
	Race	X	X	X	X	X	X	X	X
	Sex	X	X	X	X	X	X	X	X
	Relationship status	X	X	X	X	X	X	X	X
Socioeconomic Status	Education		X		X	X	X	X	X
	Income		X		X	X	X	X	X
	Medical care		X		X	X	X	X	X
Lifestyle	Smoking			X	X	X	X	X	X
	Alcohol			X	X	X	X	X	X
	BMI			X	X	X	X	X	X
	Physical act.			X	X	X	X	X	X
Comorbidities	CKD					X	X	X	X
	Diabetes					X	X	X	X
	Cardiovascular disease history					X	X	X	X
	Hypertension					X	X	X	X
	Dyslipidemia					X	X	X	X
	Atrial Fib.					X	X	X	X
Self‐Reported Health	SR health						X	X	X
Perceived Stress	Stress							X	X
Interaction	Polypharm*CKD interaction								X

Abbreviations: Act, activity; BMI, body mass index; CKD, chronic kidney disease; Fib, fibrillation; Mod, Model.

Two propensity‐adjusted models addressed confounding by indication.^33^ In these models, all candidate confounders from Table 2 were included in a multiple logistic regression (propensity) analyses that used binary polypharmacy status (defined as ≥8 total ingredients) as the dependent variable. Each participant's polypharmacy propensity was estimated, and participants’ propensities (irrespective of actual polypharmacy status) were divided into quintiles or deciles. After stratifying on propensity quintiles or deciles, a stratified, no‐interaction (hazard ratio assumed constant for all propensity quintiles/deciles) Cox proportional hazard regression used only major/minor polypharmacy as mortality predictors.

**TABLE 2:** Polypharmacy exposure status (defined as ≥8 total generic ingredients = major polypharmacy [polypharm+] vs. no/minor polypharmacy [polypharm−], 0–7 total generic ingredients) by covariate value among the entire cohort with exposure assessed and at least one follow‐up(s) (*n* = 29 627)

Covariate	Cov. Values	N	%	Mean Med Count	Polypharm+ (%)	Polypharm− (%)
Age	85+	582	1.96	5.35	23.7	76.3
	75–84	4518	15.2	5.62	26.2	73.8
	65–74	9568	32.3	5.22	23.9	76.1
	55–64	11 295	38.1	4.61	19.6	80.4
	45–54	3664	12.4	3.56	11.9	88.1
Region	Buckle^a^	6200	20.9	5.28	24.6	75.4
	Belt	10 267	34.7	5.01	22.1	77.9
	Non‐belt	13 160	44.4	4.53	18.7	81.3
Race^b^	White	17 449	58.9	4.86	20.9	79.1
	Black	12 178	41.1	4.84	21.4	78.6
Sex	Male	13 304	44.9	4.5	18.5	81.5
	Female	16 323	55.1	5.13	23.3	76.7
Education	College grad	10 325	34.9	4.34	16.3	83.7
	Some college	7928	26.8	4.86	21.3	78.7
	HS	7654	25.9	5.1	23.3	76.7
	<HS	3697	12.5	5.75	29.5	70.5
Income	≥$75 k	4684	18	3.89	13.1	86.9
	$35–$74 k	8795	33.9	4.5	17.7	82.3
	$20–$34 k	7155	27.6	5.09	23.0	77.0
	<$20 k	5331	20.5	5.7	29.2	70.8
Relationship status	Widowed	5608	19.4	5.53	26.5	73.5
	Divorced	4299	14.9	4.8	21.4	78.6
	Married	17 470	60.4	4.68	19.4	80.6
	Single	1558	5.38	4.43	19.3	80.7
Medical care	Yes	21 839	79.5	5.07	22.2	77.8
	No	5631	20.5	4.04	16.7	83.3
Insurance	Yes	27 670	93.5	4.93	21.6	78.4
	No	1931	6.52	3.67	14.3	85.7
Smoking	Current	4270	14.5	4.65	20.4	79.6
	Past	11 888	40.3	5.18	23.5	76.5
	Never	13 355	45.3	4.62	19.2	80.8
BMI (kg/m^2^)	≤18.5	312	1.06	3.96	15.1	84.9
	18.5–24.9	6971	23.7	4.01	14.2	85.8
	25.0–29.9	10 860	36.9	4.5	17.7	82.3
	≥30.0	11 284	38.3	5.7	28.5	71.5
Alcohol use	Heavy	1175	4.04	4.05	13.6	86.4
	Moderate	9673	33.3	4.26	16.1	83.9
	None	18 201	62.7	5.21	24.3	75.7
Self‐reported health	Poor	1036	3.5	9.03	59.9	40.1
	Fair	4410	14.9	6.99	41.0	59.0
	Good	10 357	35	5.23	23.4	76.6
	Very good	9027	30.5	3.91	12.2	87.8
	Excellent	4738	16	2.89	6.1	93.9
Exercise habits	None	10 041	34.4	5.66	28.1	71.9
	1–3 times/week	10 511	36	4.57	18.8	81.2
	>3 times/week	8635	29.6	4.25	15.8	84.2
CKD	Yes	3248	11.4	7.15	41.0	59.0
	No	25 123	88.6	4.52	18.3	81.7
Diabetes	Yes	6285	22	7.36	43.8	56.2
	No	22 266	78	4.16	14.9	85.1
CVD history	Yes	5219	18	7.06	40.4	59.6
	No	23 855	82	4.36	16.9	83.1
Hypertension	Yes	17 513	59.2	5.93	28.8	71.2
	No	12 050	40.8	3.27	9.9	90.1
High lipids	Yes	16 932	59.4	5.52	26.3	73.7
	No	11 594	40.6	3.9	13.7	86.3
Atrial Fib.	Yes	2543	8.79	6.85	38.3	61.7
	No	26 400	91.2	4.64	19.3	80.7

Abbreviations: CKD, chronic kidney disease; CVD, cardiovascular disease; Fib., Fibrillation; HS, high school.

For simplicity, major polypharmacy is compared to minor and no polypharmacy grouped together.

^a^ Stroke Buckle: Subset (coastal plain of Georgia, North Carolina, and South Carolina) of the stroke belt.

^b^ Race was only variable where polypharmacy chi‐square *p* value > .001.

Collinearity was assessed for the time‐on‐study models using a Statistical Analysis Software (SAS) macro.^34^ SAS 9.2 was used. The proportional hazards assumption for the time‐on‐study models was checked by constructing univariable log–log survival plots and by examining univariable model Schoenfeld residuals^35^ failure time correlations.^36^ For the age–time‐scale models, the proportional hazards assumption was assessed with Schoenfeld residuals.

## 3 RESULTS

Overall, 171 573 individual medications were transcribed during in‐home visits. The most common generics and medication classes are shown in Tables S3 and S4, respectively. Among all 30 181 participants, 21.1%, 15.8%, and 63.2% were categorized as receiving major, minor, and no polypharmacy, respectively. The cohort characteristics comparing the major polypharmacy group (polypharmacy+) to all others (polypharmacy−) are presented in Table 2. In the analytic sample, the mean age was 64.9 years, 45% were male, 41% black, 56% stroke‐belt residents, 24% with normal BMI, 11% with CKD, and 16% and 31% were in “excellent” and “very good” self‐reported health, respectively. Relative to the polypharmacy group, those with major polypharmacy (polypharmacy+) included a greater proportion of females and stroke‐belt residents, and those with less education, lower income, higher BMI, more comorbidities (CKD, hypertension, dyslipidemia, diabetes, coronary artery disease), and lower self‐reported health (Table 2). In crude analyses, older adults, blacks, males, individuals with less education or income, smokers, those with poorer self‐reported health, and those with comorbidities showed higher mortality.

**FIGURE 1:**
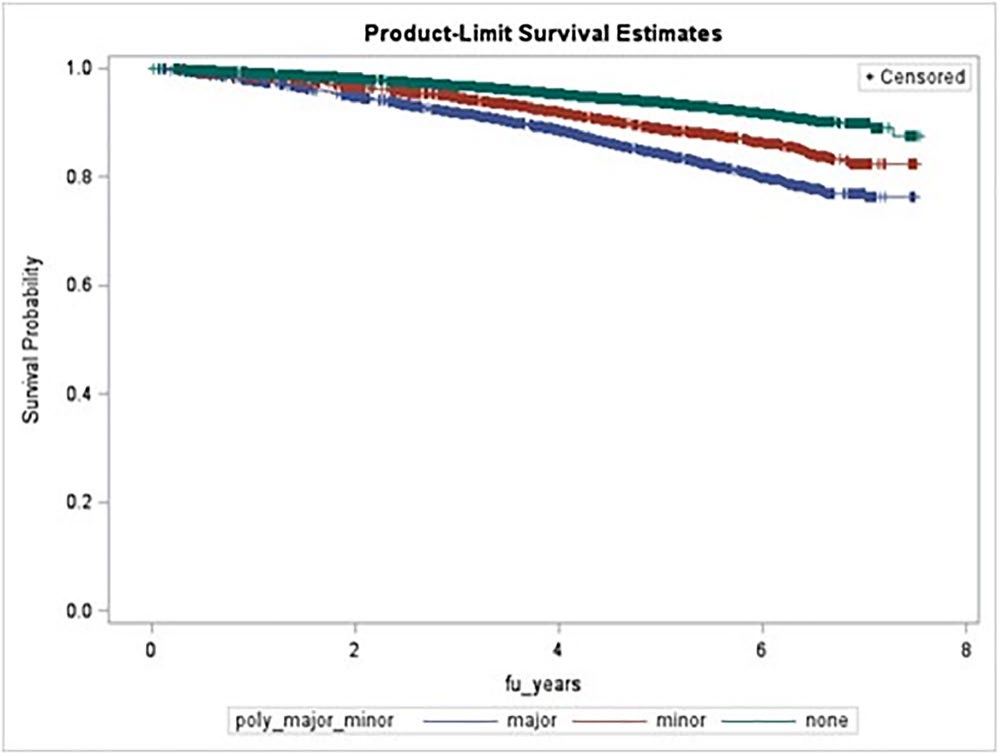
Kaplan–Meier all‐cause‐mortality plot according to polypharmacy status (no polypharmacy [green], minor [red], and major [blue]). Log rank *p* < .0001. fu_years, follow‐up years

Median follow‐up was 4.9 years; 2538 deaths occurred. As seen in the Kaplan–Meier plot (Figure 1), major polypharmacy had the highest mortality, followed by minor polypharmacy, and the no‐polypharmacy group (log‐rank *p* < .0001). In all time‐on‐study (Table 3) and age‐time‐scale (Table S1) models, major polypharmacy was significantly associated with mortality. The hazard ratio estimates ranged from 1.22 (95% CI: 1.07–1.40) to 2.35 (2.15–2.56), depending on the model. The minor polypharmacy hazard ratio estimates were smaller, ranging from 1.06 (0.92–1.22) to 1.50 (1.35–1.67).

**TABLE 3:** Multivariable analyses of the association between major and minor polypharmacy (vs. no polypharmacy) and all‐cause mortality using eight multivariable time‐on‐study models

	Time‐on‐study models	
	Major polypharm HR (95% CI)	Minor polypharm HR (95% CI)
Model 1	2.35 (2.15–2.56)	1.50 (1.35–1.67)
Model 2	2.23 (2.03–2.44)	1.48 (1.32–1.65)
Model 3	2.17 (1.97–2.38)	1.47 (1.32–1.65)
Model 4	2.09 (1.89–2.31)	1.47 (1.30–1.65)
Model 5	1.36 (1.20–1.56)	1.21 (1.05–1.39)
Model 6	1.22 (1.07–1.40)	1.14 (0.99–1.31)
Model 7	1.22 (1.07–1.40)	1.14 (0.99–1.31)
Model 8^a^	1.24 (1.06–1.45)	1.15 (0.98–1.36)

^a^ HRs for CKD = 0 individual, and CKD*Polypharm interaction terms both non‐significant (*p* **> **.70).

**FIGURE 2:**
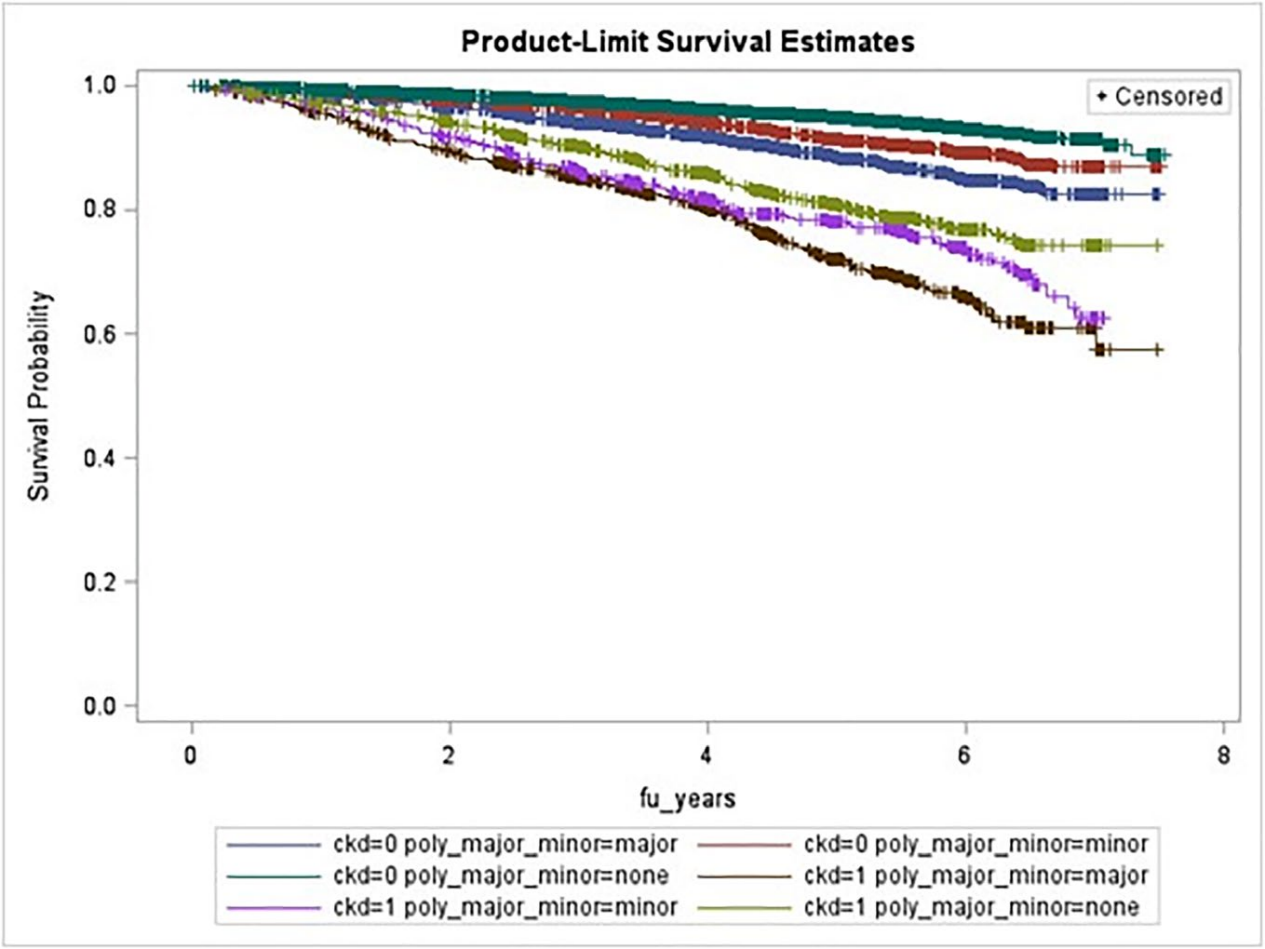
Kaplan–Meier all‐cause‐mortality plot for polypharmacy*CKD status (log rank *p*‐value < .0001). Green = CKD −, no polypharm; Red = CKD −, minor polypharm; Blue = CKD −, major polypharm; Yellow = CKD +, no polypharm; Pink = CKD +, minor polypharm; Brown = CKD +, major polypharm

Figure 2 shows survival stratified by both polypharmacy and CKD status. CKD strongly predicted mortality, and within each CKD level, there was an increased mortality going from no polypharmacy to minor to major polypharmacy. Model 8’s CKD*polypharmacy interaction terms were all non‐significant (all interaction *p* **> **.70).

The two methods of modeling time‐to‐event (age‐time‐scale and time‐on‐study) gave similar results with <3% difference across model‐specific hazard ratio estimates. The models that controlled for propensity scores using stratification gave results consistent in magnitude with models including covariates as separate terms (Table S2).

## 4 DISCUSSION

Drugs play vital and irreplaceable roles in medicine. While polypharmacy may sometimes be the standard of care, polypharmacy can occur unnecessarily and inappropriately, exposing patients to potentially serious risks and inspiring the call for “deprescribing.”^12^, ^13^, ^37^ In this longitudinal study of a racially diverse, nationwide sample of the general U.S. adult population, we found that (1) major polypharmacy was associated with mortality in all models (HR range 1.2–2.4); (2) the association was consistently less pronounced for minor polypharmacy; (3) there was no evidence that the effect of polypharmacy on mortality is modified by CKD status; and (4) propensity‐based and traditional covariate‐based analyses produced similar results.

Although imperfect and an oversimplification, polypharmacy is a well‐established concept in the clinical literature and is likely to remain so until the era of personalized medicine is realized. The numerical medication burden, even without dose or frequency consideration, is a major factor for clinicians when performing medication reconciliation and making decisions on indicated pharmacologic interventions. Aside from a placebo, no medication is universally innocuous. Given the propensity for pharmacokinetic/pharmacodynamic interactions with polypharmacy, it is a biologically meaningful variable, albeit a crude measure.

Several previous studies investigated the association between polypharmacy and mortality in a variety of populations, although large‐scale research on outpatient American adults is limited. In general, there is no literature consensus regarding the presence or absence or a polypharmacy–mortality relationship. Most published research involves European geriatrics. One factor that likely contributes to the literature's incongruous findings is that most research cannot distinguish rational, indicated as polypharmacy (e.g., using aspirin, statin, beta blocker, and angiotensin receptor blocker following a myocardial infarction), from illogical, “haphazard” polypharmacy (a type 1 diabetic who is prescribed metformin and glipizide in addition to insulin). Assuming confounding by indication could be fully controlled, then evidence‐based polypharmacy would be expected to decrease mortality (assuming the medications contributing to the polypharmacy were for high‐risk pathologies such as cardiovascular disease and diabetes and not symptomatic relief such as acetaminophen for osteoarthritis). As such, depending on the proportion of cohort members for which polypharmacy resulted from medication accumulation and not thoughtful prescribing, a positive polypharmacy–mortality association would be anticipated. Conversely, if cohort polypharmacy reflects the implementation of evidence‐based clinical guidelines, then a negative polypharmacy–mortality hazard ratio is expected. Finally, a null association would be predicted if both rational, beneficial polypharmacy and disorganized, deleterious polypharmacy were found in roughly equal proportions in the cohort.

To briefly summarize the largely international literature on polypharmacy–mortality: Jyrkka reported mixed‐polypharmacy mortality results among Finns,^38^ and Espino found a positive association among Mexican‐Americans.^39^ Iwata reported higher 1‐year mortality among Japanese elderly polypharmacy users following hospital discharge.^40^ Incalzi reported higher in‐hospital mortality among Italian polypharmacy patients.^41^ Richardson reported higher 2‐year mortality in older UK polypharmacy users.^42^ Spanish,^43^ French,^44^ Italian,^45^ Chinese,^46^ Brazilian,^47^ and New Zealand^48^ geriatric research also reported increased mortality among polypharmacy patients. Conversely, Wauters found no polypharmacy–mortality association in a small geriatric Belgian cohort^49^ and, furthermore, report an association between geriatric medication underuse and mortality.^50^ Schlesinger found no polypharmacy–mortality relationship in a small Israeli nursing home cohort^51^; Bonaga reported no mortality association in non‐frail Spanish geriatrics^52^; and Wimmer, defining polypharmacy as a continuous variable, found no increased mortality hazard for each additional medication used among Swedish geriatrics.^53^ Similarly, in two Italian studies, Pozzi reported no polypharmacy–mortality association,^54^ and Nobili observed no association between polypharmacy and in‐hospital mortality among hospitalized elderly patients.^55^

Regarding the limited prior exploration of the polypharmacy–mortality relation in Americans, Secora used the Atherosclerosis Risk in Communities (ARIC) cohort and found an overall similar polypharmacy dose–response association with mortality, along with a lack‐of‐effect modification by CKD status.^56^ However, the REGARDS cohort is much larger than ARIC and has a national scope, we defined polypharmacy differently, and we used a broader range of models. Finally, the consistency of results with our propensity‐matched analyses contrasts with Schöttker's analyses of polypharmacy and mortality in German adults where their original multivariate‐adjusted association was lost after also controlling for propensity score.^57^

The finding of significant hazard ratios for major polypharmacy after adjusting for potential confounders in all models and the graded polypharmacy–mortality relationship ([major polypharmacy hazard] > [minor polypharmacy hazard]) is biologically plausible. Conversely, we found little support for the a priori hypothesis that polypharmacy would be more harmful among those with CKD. It is important to recognize that the inter‐relation between CKD and polypharmacy may be complex and not sufficiently described by a simple dichotomized CKD*polypharmacy interaction. For example, polypharmacy may decrease mortality in individuals with more severe kidney disease for whom a regimen of multiple drugs may be beneficial. Alternatively, polypharmacy may increase mortality in individuals with mild renal impairment who, perhaps unaware of diminished drug clearance, may suffer greater toxicity. We defined CKD in the usual way: dialysis or glomerular filtration rate <60 mL/min/1.73 m^2^. This corresponds to CKD stage 3 and greater, although a limitation is the lack of albuminuria consideration, which is known to be a key moderator of CKD‐related cardiovascular disease.^58^ Our analysis has important strengths. Rigorous exposure and outcome assessments minimized misclassification. Many potential confounders were measured. The large sample size and long follow‐up provided ample statistical power. Moreover, the sample was generated from the general, biracial population of community‐dwelling American adults, with minimal exclusion criteria, suggesting that the results are reasonably generalizable.

While the low percentage (<3%) of participants who dropped out of the study annually may limit selection bias, the duration of follow‐up (median 4.9 years) may mean that the total loss to follow‐up is non‐trivial. Nevertheless, some event‐free survival time information was available for >98% of the cohort. Our polypharmacy definition is similar to many in the literature, but there is no “gold standard” definition.^59^ Longer follow‐up is available in the REGARDS data. However, these data were not utilized, as corresponding longitudinal polypharmacy assessment is unavailable and the likelihood of misclassification would increase with longer follow‐up. Confounding by indication, the fact that those taking and not taking medications are systematically different (beyond drug use), and residual confounding presented additional methodological challenges. Data on many potential confounders were collected (and the number of events sufficient for large models), so residual confounding may be limited, as well as by the propensity score–based analyses.

Absent an established biological mechanism linking polypharmacy and all‐cause mortality, it is possible that a model's supposed “confounders” may function as polypharmacy‐based mediators acting in either a causal or a preventative outcome pathway. Because of the complex exposure patterns (billions of drug combinations) and numerous biological processes converging in death, it is difficult a priori to distinguish confounders from mediators.

As such, given the heterogeneous biological nature of both exposure and outcome, selecting an “optimal” model that accounts for the underlying pharmacology is challenging; the results are conditional on the models. We addressed this problem by conducting analyses comparing the “full” model (with many possible confounders) to a series of reduced models.

Additional important limitations include that no information on medication indication, dose, or use frequency/use duration was collected. Also, it is implicitly assumed that one baseline medication measurement accurately represents pharmacological burden throughout follow‐up. The polypharmacy metric did not distinguish eye drops/skin creams from pills/injectables when aggregating total generic ingredients. To the extent that eye drops/creams may not enter the systemic circulation, they would not be expected to contribute to mortality as much as oral/injectable agents. Additionally, medication misclassification is possible at multiple stages—incompletely assembled medications, medication transcription mistakes, electronic database scanning errors, and generic name assignment.

Clinicians recognize that medication reconciliation is critical to good care. However, with polypharmacy, accurate medication regimen accounting can take 10 min. This temporal outlay is not feasible for appointments as brief as 15–20 min. While this study provides no novel strategy to mitigate polypharmacy risks, it highlights the traditional teaching of the medication history's primacy, which offers many well‐established benefits (cost, side effects, and quality of life) with de‐prescribing medications without clear indication. We envision this research as hypothesis generating and a reminder to providers that an accurate medication reconciliation (not assuming the pre‐populated electronic medical record list is accurate) is foundational to clinical care and will often change disease management.

## 5 CONCLUSIONS

We found a polypharmacy and all‐cause mortality association. As hypothesized, mortality was related to polypharmacy degree; however, unexpectedly, no CKD effect modification was observed. Further research is warranted to understand the impact of drug dosages and the relative contributions of different drug classes to the observed polypharmacy–mortality relationship. The specificity of the biological pathway(s) (e.g., refined pharmacological exposure beyond simple medication count) and exploration of potential CKD‐based polypharmacy vulnerability (or therapeutic opportunity) merit further investigation.

## DISCLOSRE

The authors have no conflicts of interest to declare.

## DATA VERIFICATION

WC had full access to all study data and takes responsibility for the integrity of the data and the accuracy of the data analysis. All authors confirm a role in the manuscript.

## Supporting information

Data S1
Table S1–S4

## Data Availability

The data that support the findings of this study are available on request from the corresponding author. The data are not publicly available due to privacy or ethnical restrictions.
