# Supplementary material for: Polypharmacy and mortality association by chronic kidney disease status: The REasons for Geographic And Racial Differences in Stroke Study
Source: Pharmacol Res Perspect. 2021 Aug 2;9(4):e00823. doi: 10.1002/prp2.823 (PMC8328192; doi:10.1002/prp2.823)
Supplement: Supplementary file 1 — Data S1 Table S1–S4 [file PRP2-9-e00823-s001.docx]

**Supplementary Text**:

**Sample Sizes and Medication-Use Assumptions for 0.29% of cohort:** The total cohort size is 30,183. However, 30,157 cohort members were present in the medication file (26 (0.09%) “apparently” had missing medication forms). However, for 25 of those, other in-home variables were collected, and it was assumed that the medication form was left blank simply because no medications were taken. These 25 were classified as non-medication users. The one individual for whom no other in-home variables were recorded was assumed to have a missing medication form and excluded from analysis. Amongst the 30,157 cohort members present in the medication form file, 63 (0.21%) were missing all medication form variables. However, for 62 of these individuals, other in-home variables were collected, and the reason for the missing medication form data was assumed to be that it was left blank because no medications were being taken. As such, these 62 were also classified as non-medication users. Finally, 1 cohort member was present in the medication file but was missing all medication form and in-home variables. As such, she was excluded from analyses. Thus, the total polypharmacy n = 30,181 (two cohort members excluded for missing data). However, 554 of these 30,181 lacked any outcome follow-up vital status or follow-up time and were thus excluded from analyses. Thus, the final analytical N = 29,627.

**PH Assumption Testing:** No strongly nonparallel univariable log-log survival vs. log(follow-up time) plots were observed for any variable (the plots did sometimes cross, but for limited data portions). Many of the log-log survival plots were linear, suggesting a possible univariable Weibull survival distribution[^35^](#_ENREF_35). However, for multivariable models, Cox PH models (without the Weibull survival assumption) were utilized.

For the univariable time-on-study Schoenfeld residual correlations, all variables had a correlation p > 0.05 or a correlation coefficient absolute value < 0.07. Because of the small (albeit sometimes statistically significant) absolute correlations, the PH assumption was considered reasonable for all variables. Bivariable (including one time-dependent term) extended-Cox models were constructed; some had statistically significant time-dependent terms.

For the time-on-study models, the PH assumption was deemed reasonable for all variables considered one-at-a-time. For the univariable age-time-scale models, the PH assumption was deemed reasonable (Schoenfeld Residual correlation p > 0.05 or correlation coefficient absolute value < 0.15) for all variables considered one-at-a-time.

**Definitions of Potential Confounders**:

1. **Medical Care**: Cohort member response to “Do you have any kind of healthcare coverage such as health insurance, an HMO, or a government plan like Medicare or Medicaid?”
2. **Self-Reported Health**: Cohort member multiple choice response to “in general, would you say that your health is…”
3. **Diabetes**: taking anti-hyperglycemic or fasting glucose ≥ 126 mg/dl or non-fasting glucose ≥ 200 mg/dl
4. **Cardiovascular Disease History**: history of prior myocardial infarction, coronary bypass/angioplasty/stenting, or EKG evidence of old infarct OR reported prior stroke
5. **Hypertension**: in-home visit systolic blood pressure > 140 mmHg or diastolic > 90 mmHg or self-reported use of anti-hypertensive
6. **Hyperlipidemia**: total cholesterol ≥ 240 mg/dl, low density lipoprotein ≥ 160 mg/dl, high density lipoprotein ≤ 40 mg/dl, or use of a cholesterol lowering medication
7. **Atrial Fibrillation**: based on self-report or EKG
8. **Stress**: Cohen’s Perceived Stress Scale calculated from computer-assisted telephone interview questions

**Supplementary Tables**:

**Supplementary Table A:** Association between Major and Minor Polypharmacy (vs. no polypharmacy) and All-Cause Mortality Using Eight Distinct **Age-Time-Scale** (Conditioning on Age at Study Entry and Stratifying by Birth Cohort) Models.

|  | **AGE-TIME-SCALE MODELS** |  |  |
| --- | --- | --- | --- |
|  | **Major Polypharm**  **HR (95% CI)** | **Minor Polypharm**  **HR (95% CI)** |  |
| **Model 1** | 2.31 (2.11-2.52) | 1.48 (1.33-1.65) |  |
| **Model 2** | 2.20 (2.01-2.42) | 1.46 (1.31-1.63) |  |
| **Model 3** | 2.12 (1.93-2.33) | 1.44 (1.29-1.61) |  |
| **Model 4** | 2.06 (1.86-2.27) | 1.44 (1.28-1.62) |  |
| **Model 5** | 1.38 (1.21-1.58) | 1.20 (1.05-1.38) |  |
| **Model 6** | 1.23 (1.07-1.40) | 1.13 (0.98-1.30) |  |
| **Model 7** | 1.23 (1.07-1.40) | 1.13 (0.98-1.30) |  |
| **Model 8*** | 1.23 (1.05-1.45) | 1.14 (0.97-1.34) |  |
| *HRs for CKD=0 individual, CKD*Polypharm interaction terms both non-significant (p>0.75) | | | |

**Supplementary Table B**: Propensity-Stratified Models (Age-Time-Scale and Time-On-Study) and Their Estimated Major and Minor Polypharmacy HRs (vs. no polypharmacy)

|  | **Major Polypharm HR (95% CI)** | **Minor Polypharm HR**  **(95% CI)** |
| --- | --- | --- |
| **Quintile Stratified, Age-Time-Scale** | 1.30 (1.14-1.48) | 1.08 (0.94-1.24) |
| **Decile Stratified, Age-Time-Scale** | 1.22 (1.07-1.40) | 1.06 (0.92-1.22) |
| **Quintile Stratified, Time-on-Study** | 1.31 (1.15-1.50) | 1.13 (0.98-1.30) |
| **Decile Stratified, Time-on-Study** | 1.23 (1.08-1.41) | 1.11 (0.96-1.28) |

**Supplementary Table C**: Most common REGARDS generic medications

| **generic name** | **prevalence [%]** |
| --- | --- |
| aspirin | 34.5 |
| hydrochlorothiazide | 21.6 |
| acetaminophen | 13.4 |
| atorvastatin | 12.8 |
| simvastatin | 11.1 |
| lisinopril | 10.5 |
| amlodipine | 10.2 |
| metoprolol | 9.9 |
| levothyroxine | 9.9 |
| metformin | 9.2 |
| atenolol | 7.9 |
| furosemide | 7.6 |
| ibuprofen | 6.5 |
| triamterene | 5.1 |
| valsartan | 5.0 |
| naproxen | 4.7 |
| clopidogrel | 4.6 |
| benazepril | 4.3 |
| esomeprazole | 4.1 |
| conjugated estrogens | 4.0 |
| omeprazole | 4.0 |

**Supplementary Table D**: Most common REGARDS generic medication classes

| **MEDICATION CLASS** | **PREV [%]** |
| --- | --- |
| platelet aggregation inhibitor | 36.6 |
| salicylate | 34.8 |
| statin | 31.8 |
| angiotensin converting enzyme Inhibitor | 23.6 |
| thiazide diuretic | 23.0 |
| cardio-selective beta blocker | 18.8 |
| Ca channel blocker | 17.8 |
| NSAID | 15.5 |
| proton pump inhibitor | 15.4 |
| misc analgesic | 14.3 |
| angiotensin receptor blocker | 14.1 |
| antihistamine | 12.0 |
| antiarrhythmic agent | 12.0 |
| thyroid drug | 10.2 |
| non-sulfonylurea | 9.2 |
| SSRI | 8.6 |
| loop diuretic | 8.5 |
| sulfonylurea | 8.4 |
| narcotic analgesic | 7.3 |
| estrogen | 7.1 |
| potassium sparing diuretic | 6.6 |
| inhaled corticosteroid | 6.4 |
| cox II Inhibitor | 6.2 |
